# A Public Health Paradox: The Women Most Vulnerable to Malaria Are the Least Protected

**DOI:** 10.1371/journal.pmed.1002014

**Published:** 2016-05-03

**Authors:** Raquel González, Esperança Sevene, George Jagoe, Laurence Slutsker, Clara Menéndez

**Affiliations:** 1 ISGlobal, Barcelona Ctr. Int. Health Res. (CRESIB), Hospital Clínic- Universitat de Barcelona, Barcelona, Spain; 2 Manhiça Health Research Center (CISM), Manhiça, Mozambique; 3 Eduardo Mondlane University, Faculty of Medicine, Maputo, Mozambique; 4 Medicines for Malaria Venture, Geneva, Switzerland; 5 Division of Parasitic Diseases and Malaria, Center for Global Health, Centers for Disease Control and Prevention, Atlanta, Georgia, United States of America

## Abstract

Raquel Gonzalez and colleagues highlight an urgent need to evaluate antimalarials that can be safely administered to HIV-infected pregnant women on antiretroviral treatment and cotrimoxazole prophylaxis.

**Summary Points**

- African HIV-infected pregnant women are the most vulnerable population group to malaria infection.
- Paradoxically, these women are also the least protected against malaria due to fear of potential interactions between antiretroviral and antimalarial drugs.
- Action is urgently needed to evaluate antimalarials that can be safely administered to HIV-infected pregnant women on antiretroviral treatment and cotrimoxazole prophylaxis.

## Who Are the Women Most Vulnerable to Malaria?

Sub-Saharan Africa (SSA) is considered the be the centre of the global HIV epidemic with the highest prevalence and incidence of HIV infection globally and where women account for approximately 57% of all people living with HIV [1]. SSA also concentrates the greatest burden of malaria. In this region, approximately 30 million pregnancies occur annually in areas of intense *Plasmodium falciparum* transmission, and HIV-infected women are known to be the most vulnerable to malaria infection [2,3].

For reasons not completely understood, pregnant women are particularly vulnerable to malaria, with more frequent and higher density infections than nonpregnant women. Malaria in pregnancy is associated with significant maternal and infant morbidity and mortality [4]. Of note, an estimated 20 million HIV-infected individuals in SSA live in malaria endemic areas, and over 12 million are women of reproductive age [1]. In addition, approximately one million pregnancies each year are complicated by coinfection with malaria and HIV in SSA [1]. As a group, women in this region are the most vulnerable to HIV infection due to biological and sociocultural factors [3]. As with malaria, maternal HIV infection increases the risk of miscarriage, stillbirth, and other adverse birth outcomes [5]. The interaction between the two infections is particularly deleterious in pregnancy. HIV increases the severity of malaria infection and disease, and malaria infection increases HIV viral load, which in some studies has been shown to increase the risk of mother-to-child transmission of HIV (MTCT-HIV)[6].

## Are Current Malaria Control Strategies Sufficiently Effective for HIV-Infected Pregnant Women?

The current WHO recommendation for control of malaria in pregnant women living in stable transmission areas relies on both the administration of Intermittent Preventive Treatment with sulfadoxine-pyrimethamine (IPTp-SP) beginning as early as possible in the second trimester and at every scheduled antenatal care (ANC) visit thereafter, along with the use of insecticide-treated bed nets (ITNs) [7]. However, in HIV-infected women, IPTp-SP is contraindicated to avoid the potentially serious drug interactions with concomitant cotrimoxazole prophylaxis (CTXp), which is currently recommended in all HIV-infected pregnant women to prevent opportunistic infections [8]. Thus, even though IPTp-SP is a life-saving and highly cost-effective intervention, it cannot be used in the most vulnerable group, HIV-infected women [9,10].

Because of the proven antimalarial effect of cotrimoxazole, it has been assumed that CTXp would provide effective malaria prevention in HIV-infected pregnant women [11]. However, evidence to support this assumption is sparse and requires additional confirmation [12]. In addition, programmatic effectiveness of CTXp may be suboptimal due to the challenges of adherence to a daily regimen of indefinite duration [13]. In a recent study, the addition of an efficacious antimalarial drug (mefloquine) to CTXp in HIV-infected pregnant women improved malaria prevention as evidenced by reductions in peripheral parasitemia and placental infection, as well as improvement in overall maternal health with decreased hospital admissions [6]. However, mefloquine prophylaxis was not well tolerated, and importantly, was found to be associated with both an increased maternal HIV viral load at delivery and risk of MTCT-HIV. In this study, most of the nonobstetric admissions among HIV-infected women were due to infectious diseases, which are known to be an important cause of maternal death in these women [6]. The immunosuppressive effect of malaria is well documented; therefore, the effective prevention of malaria could help to reduce the risk of opportunistic infections. The effect of malaria as a risk factor for death in HIV-infected individuals is increasingly recognised, and it has been recently reported among HIV-infected children in Malawi [14].

Unfortunately, the assumption that HIV-infected pregnant women are well protected against malaria by CTXp has curtailed evaluation of other drugs for this purpose [15,16]. Of note, prevention of MTCT-HIV through lifelong administration of antiretroviral therapy (ART) to HIV-infected pregnant women (termed “option B+”), as well as the recent WHO recommendation to initiate ART for every HIV-infected individual regardless of the CD4 cell count (“treat all”), should lead to an increase in the survival and therefore number of HIV-infected women of reproductive age [8,17]. This may lead to an increase in the number of women who become pregnant and are exposed to malaria in endemic areas. In this context, the lack of specifically designed studies to evaluate additional malaria prevention strategies in this special population means that the most vulnerable women are also the least protected. Thus, studies are needed in HIV-infected pregnant women in endemic areas in SSA to evaluate improved malaria prevention tools, including alternative antimalarial drugs. These studies should include (or be preceded by) careful assessment of potential pharmacological and safety interactions between antimalarial and antiretroviral drugs.

## What Are the Challenges and the Way Forward?

Pregnancy itself increases the complexity of the clinical management of the malaria-HIV coinfection by reducing the therapeutic options and by altering the function of drug-metabolizing enzymes and drug transporters in a gestational-stage and tissue-specific manner [18,19]. Recent pharmacokinetic studies indicate that a significant reduction in systemic exposure to some antiretroviral and antimalarial drugs may occur when administered concomitantly, raising concerns about an increased risk of treatment failures and/or safety issues [20,21]. These disturbing results point to the need for further investigation to evaluate the clinical relevance of these drug–drug interactions in pregnancy.

More generally, as new policies such as “treat-all” and “option B+” are scaled up, new and complex public health challenges may appear due to the increasing number of HIV-infected people who would be exposed to ART. For example, it would be important to implement active pharmacovigilance systems in some sentinel sites to monitor possible drug-related adverse events, as well as to reinforce the health system to guarantee the sustainability of ART administration to all HIV-infected individuals and long-term treatment adherence to prevent the appearance of viral mutants of resistance. Moreover, in malaria-endemic areas, HIV-infected individuals—in addition to being more likely to receive antimalarial drugs for treatment due to their increased risk of malaria—may also be receiving these drugs for prevention; examples include seasonal malaria chemoprevention or mass drug administration during malaria elimination efforts. Thus, the problem of malaria–HIV coinfection needs to be revisited to take into account the new context and evolving intervention strategies for both diseases.

## Abbreviations

ANC: antenatal care
CTXp: cotrimoxazole prophylaxis
IPTp-SP: Intermittent Preventive Treatment with sulfadoxine-pyrimethamine
ITN: insecticide-treated bed net
MTCT-HIV: mother-to-child transmission of HIV
SSA: sub-Saharan Africa
